# Assessment of Care Cascades Following Low-Value Prostate-Specific Antigen Testing Among Veterans Dually Enrolled in the US Veterans Health Administration and Medicare Systems

**DOI:** 10.1001/jamanetworkopen.2022.47180

**Published:** 2022-12-01

**Authors:** Aimee N. Pickering, Xinhua Zhao, Florentina E. Sileanu, Elijah Z. Lovelace, Liam Rose, Aaron L. Schwartz, Allison H. Oakes, Jennifer A. Hale, Loren J. Schleiden, Walid F. Gellad, Michael J. Fine, Carolyn T. Thorpe, Thomas R. Radomski

**Affiliations:** Center for Health Equity Research and Promotion, Veterans Affairs Pittsburgh Healthcare System, Pittsburgh, Pennsylvania; Division of General Internal Medicine, University of Pittsburgh School of Medicine, Pittsburgh, Pennsylvania; Center for Health Equity Research and Promotion, Veterans Affairs Pittsburgh Healthcare System, Pittsburgh, Pennsylvania; Center for Health Equity Research and Promotion, Veterans Affairs Pittsburgh Healthcare System, Pittsburgh, Pennsylvania; Center for Health Equity Research and Promotion, Veterans Affairs Pittsburgh Healthcare System, Pittsburgh, Pennsylvania; Health Economics Resource Center, Veterans Affairs Palo Alto Healthcare System, Palo Alto, California; Center for Health Equity Research and Promotion, Crescenz Veterans Affairs Medical Center, Philadelphia, Pennsylvania; Division of General Internal Medicine, Department of Medical Ethics Health Policy, University of Pennsylvania, Philadelphia; Center for Health Equity Research and Promotion, Veterans Affairs Pittsburgh Healthcare System, Pittsburgh, Pennsylvania; Trilliant Health, Birmingham, Alabama; Center for Health Equity Research and Promotion, Veterans Affairs Pittsburgh Healthcare System, Pittsburgh, Pennsylvania; Center for Health Equity Research and Promotion, Veterans Affairs Pittsburgh Healthcare System, Pittsburgh, Pennsylvania; Center for Health Equity Research and Promotion, Veterans Affairs Pittsburgh Healthcare System, Pittsburgh, Pennsylvania; Division of General Internal Medicine, University of Pittsburgh School of Medicine, Pittsburgh, Pennsylvania; Center for Health Equity Research and Promotion, Veterans Affairs Pittsburgh Healthcare System, Pittsburgh, Pennsylvania; Division of General Internal Medicine, University of Pittsburgh School of Medicine, Pittsburgh, Pennsylvania; Center for Health Equity Research and Promotion, Veterans Affairs Pittsburgh Healthcare System, Pittsburgh, Pennsylvania; Division of Pharmaceutical Outcomes and Policy, Eshelman School of Pharmacy, University of North Carolina, Chapel Hill; Center for Health Equity Research and Promotion, Veterans Affairs Pittsburgh Healthcare System, Pittsburgh, Pennsylvania; Division of General Internal Medicine, University of Pittsburgh School of Medicine, Pittsburgh, Pennsylvania

## Abstract

**IMPORTANCE:**

Older US veterans commonly receive health care outside of the US Veterans Health Administration (VHA) through Medicare, which may increase receipt of low-value care and subsequent care cascades.

**OBJECTIVE:**

To characterize the frequency, cost, and source of low-value prostate-specific antigen (PSA) testing and subsequent care cascades among veterans dually enrolled in the VHA and Medicare and to determine whether receiving a PSA test through the VHA vs Medicare is associated with more downstream services.

**DESIGN, SETTING, AND PARTICIPANTS:**

This retrospective cohort study used VHA and Medicare administrative data from fiscal years (FYs) 2017 to 2018. The study cohort consisted of male US veterans dually enrolled in the VHA and Medicare who were aged 75 years or older without a history of prostate cancer, elevated PSA, prostatectomy, radiation therapy, androgen deprivation therapy, or a urology visit. Data were analyzed from December 15, 2020, to October 20, 2022.

**EXPOSURES:**

Receipt of low-value PSA testing.

**MAIN OUTCOMES AND MEASURES:**

Differences in the use and cost of cascade services occurring 6 months after receipt of a low-value PSA test were assessed for veterans who underwent low-value PSA testing in the VHA and Medicare compared with those who did not, adjusted for patient- and facility-level covariates.

**RESULTS:**

This study included 300 393 male US veterans at risk of undergoing low-value PSA testing. They had a mean (SD) age of 82.6 (5.6) years, and the majority (264 411 [88.0%]) were non-Hispanic White. Of these veterans, 36 459 (12.1%) received a low-value PSA test through the VHA, which was associated with 31.2 (95% CI, 29.2 to 33.2) additional cascade services per 100 veterans and an additional $24.5 (95% CI, $20.8 to $28.1) per veteran compared with the control group. In the same cohort, 17 981 veterans (5.9%) received a PSA test through Medicare, which was associated with 39.3 (95% CI, 37.2 to 41.3) additional cascade services per 100 veterans and an additional $35.9 (95% CI, $31.7 to $40.1) per veteran compared with the control group. When compared directly, veterans who received a PSA test through Medicare experienced 9.9 (95% CI, 9.7 to 10.1) additional cascade services per 100 veterans compared with those who underwent testing within the VHA.

**CONCLUSIONS AND RELEVANCE:**

The findings of this cohort study suggest that US veterans dually enrolled in the VHA and Medicare commonly experienced low-value PSA testing and subsequent care cascades through both systems in FYs 2017 and 2018. Care cascades occurred more frequently through Medicare compared with the VHA. These findings suggest that low-value PSA testing has substantial downstream implications for patients and may be especially challenging to measure when care occurs in multiple health care systems.

## Introduction

Nearly all older US veterans enrolled in the US Veterans Health Administration (VHA) are also enrolled in Medicare and may receive care in VHA and non-VHA settings.^1-3^ Although dual health care system use can increase timely access to care, veterans who receive care through both the VHA and Medicare are at risk of overusing health services, incurring increased costs, and experiencing worse health outcomes compared with those who receive care within the VHA alone.^4-8^

Low-value care—the use of a health service whose costs or harms exceeds its benefits—is common within the VHA and Medicare and may result in unnecessary downstream tests, treatments, and visits, referred to as a low-value care cascade.^9-16^ Low-value prostate-specific antigen (PSA) testing to screen for prostate cancer in older adults is one of the most common low-value health services delivered in the VHA, affecting as many as 1 in 6 male veterans aged 75 years or older.^17,18^ Low-value PSA testing may result in the overdiagnosis of prostate cancer and subject patients to care cascades such as those related to receiving a prostate biopsy.^19-21^

Because rates of low-value service use in Medicare have been found to exceed those in the VHA, veterans dually enrolled in the VHA and Medicare may be at increased risk of experiencing low-value PSA testing and subsequent care cascades.^17,22^ However, the degree to which VHA-enrolled veterans receive low-value PSA testing through Medicare and the extent to which it results in care cascades is unknown. Without capturing veterans’ receipt of low-value PSA testing and subsequent care cascades both within and outside VHA through Medicare, the overall effect of low-value PSA testing and multiple health care system use on veterans has likely been underestimated.

Therefore, the objectives of this study were (1) to characterize the frequency, cost, and source of low-value PSA testing and associated care cascades among veterans dually enrolled in the VHA and Medicare and (2) to determine whether receiving an initial low-value PSA test in the VHA vs Medicare was associated with a greater degree of downstream cascade services.

## Methods

### Study Design and Data Sources

We conducted a retrospective cohort study of US veterans continuously enrolled in the VHA and fee-for-service Medicare in fiscal years (FYs) 2017 and 2018. This study was deemed exempt by the US Department of Veterans Affairs (VA) Pittsburgh Health System Institutional Review Board, which also granted a waiver of informed consent and HIPAA (Health Insurance Portability and Accountability Act) authorization. The study followed the Strengthening the Reporting of Observational Studies in Epidemiology (STROBE) reporting guideline.

We linked national patient data from the VHA and the US Centers for Medicare and Medicaid Services (CMS). Sources of VHA data included the following: the VA Corporate Data Warehouse to identify patient sociodemographic characteristics, comorbidities, *International Statistical Classification of Diseases, 10th edition* (ICD-10) codes, and *Current Procedural Terminology* (CPT) codes; the Area Resource File and VHA Support Service Center files to identify facility-level covariates; and the VA Planning Systems Support Group database to obtain patient driving distance to the nearest VHA facility. The CMS data included the Beneficiary Summary File for enrollment and sociodemographic data and the MEDPAR (Medicare Provider Analysis and Review), Inpatient, Skilled Nursing Facility, Outpatient, Home Health Agency, Hospice, Durable Medical Equipment, and Carrier files for health service utilization data.

### Study Cohort

From a preexisting national cohort of 5 242 301 VHA beneficiaries,^17^ we identified veterans aged 65 years or older continuously enrolled in the VHA and fee-for-service Medicare during FYs 2017 to 2018. We further limited the sample to men aged 75 years or older with at least 1 outpatient primary care or urology visit in the VHA or Medicare in the first half of FY 2018. To exclude veterans in this group for whom PSA testing may not be of low value, we used VHA and Medicare data to exclude those with a history of prostate cancer, radiation therapy, androgen deprivation therapy, prostatectomy, elevated PSA, or a urology visit in the year before the index date, which corresponded to the date of the first PSA test in FY 2018 for those who underwent low-value PSA testing or the date of the first primary care or urology outpatient visit in FY 2018 for those who did not. The remaining cohort represents those veterans for whom PSA testing would most likely be considered low value based on established guidelines and prior literature (eTable 1 in Supplement 1).^18,22-25^

### Low-Value PSA Testing and Control Groups

Among veterans in the study cohort, we identified those who received a low-value PSA test through either the VHA or Medicare during the first half of FY 2018. We chose this time period to allow for cascade services to be examined 6 months after the low-value PSA test in the remainder of FY 2018.^9,26^ For veterans undergoing more than 1 PSA test, we defined the first test as the index service. We labeled those veterans who received a PSA test within the VHA as the VHA PSA group and those who received a PSA test through Medicare as the Medicare PSA group. Our control group consisted of the remaining veterans in the study cohort who had not undergone PSA testing.

### Frequency, Cost, and Source of Potential Cascade Services

Our outcomes consisted of potential cascade services, defined as additional services that veterans experienced following a low-value PSA test. Potential cascade services consisted of (1) additional PSA tests, (2) non–urology outpatient visits for prostate cancer or elevated PSA, (3) urology visits, (4) prostate imaging, (5) prostate biopsy, (6) androgen deprivation therapy, (7) prostatectomy, and (8) radiation treatment (eTable 1 in Supplement 1). These services were defined by practicing clinicians on the research team, informed by their clinical knowledge and findings in relevant literature.^21^

For the PSA groups, we identified outcomes that occurred within 6 months from the date of the low-value PSA test using claims from both the VHA and Medicare. For the control group, we identified outcomes from the date of the first primary care or urology visit of FY 2018 occurring within either the VHA or Medicare. To capture services such as surgical procedures that may take several months to schedule without overcapturing services that may not be attributable to the initial low-value PSA test (eg, a urology visit for another indication), we chose an outcome period of 6 months given wait times within the VHA.^26^

We computed costs by applying VA Health Economics Resource Center value estimates to CPT codes associated with the initial PSA test or cascade services. These validated estimates represent hypothetical reimbursement based on mean national Medicare and private-sector reimbursement rates and incorporate applicable facility fees.^27^ These costs did not include other associated services such as venipuncture or patient payment.

### Patient- and Facility-Level Covariates

We established covariates using VHA and Medicare data from FY 2017. Patient-level covariates included age, race and ethnicity (Hispanic, non-Hispanic Black, non-Hispanic White, other racial or ethnic minority group, or multiracial), VA priority group, driving distance to the nearest VHA facility, and the total number and presence of specific Elixhauser comorbidities.^28^ Race and ethnicity data were captured by self-report at the time of enrollment. We also assigned veterans to the parent-station VA medical center where they received most of their outpatient care in FY 2017 and determined the facility’s corresponding academic affiliation, census region, rurality, facility complexity level,^29^ and size (based on outpatient visit volume). We chose a broad set of patient- and facility-level covariates including race and ethnicity in order to create balance among the PSA groups and control group. We imputed missing values for covariates (≤5% on any individual variable) using single imputation by chained equations.^30^

### Statistical Analysis

We determined the overall and individual counts of potential cascade services per 100 veterans during the 6-month outcome period for all groups. We then used 3 separate negative binomial models that included stabilized inverse probability of treatment weights (IPTWs) and robust variance estimates to compare the adjusted rates of potential cascade services in the VHA PSA group vs the control group, the Medicare PSA group vs the control group, and the VHA PSA group vs the Medicare PSA group. We used stabilized IPTWs to create balance between groups with regard to patient- and facility-level covariates and applied robust variance estimates to adjust for clustering effects at the VA facility level. Additional details on how IPTWs were generated are provided in the eMethods in Supplement 1. We conducted a sensitivity analysis including only veterans older than the mean age of 83 years to test the robustness of our findings.

We also determined the overall and individual cost of potential cascade services during the 6-month outcome period, presented as absolute total cost and mean unadjusted cost per veteran. We then used weighted linear regression models to compare the adjusted cost of potential cascade services in the VHA PSA group vs the control group, the Medicare PSA group vs the control group, and the VHA PSA group vs the Medicare PSA group. To account for the highly skewed nature of the cost data, which contains many 0 values, we estimated SEs and 95% CIs using a nonparametric bootstrap approach, which is a more flexible alternative for comparing arithmetic means despite nonnormality of distributions.^31^

For veterans who underwent low-value PSA testing, we determined unadjusted counts of the individual potential cascade services by source of care (VHA or Medicare). We conducted all analyses using SAS, version 7.1 (SAS Institute), and Stata, version 15.1 (StataCorp). Data were analyzed from December 15, 2020, to October 20, 2022.

## Results

There were 5 242 301 VHA-enrolled veterans in the overall cohort. Their mean (SD) age was 62.5 (16.0) years, 91.7% were men, and 68.0% were non-Hispanic White. Among the overall cohort, we identified 1 415 334 veterans (27.0%) aged 65 years or older who were dually enrolled in VHA and Medicare in FYs 2017 to 2018. Among these veterans, we identified 520 045 male veterans aged 75 years or older with at least 1 outpatient primary care or urology visit in the first half of FY 2018. After exclusion diagnoses and procedures were applied, 300 393 veterans were included in our final cohort (Table 1).

Among the 300 393 included veterans, 36 459 (12.1%) underwent PSA testing within the VHA, 17 981 (5.9%) underwent PSA testing through Medicare, and 245 953 (82.0%) did not undergo PSA testing (Figure). After the IPTWs were applied, the standardized mean differences were less than 0.1 for all patient- and facility-level covariates, indicating appropriate balance for all covariates through use of our propensity score models (eTables 2-4 in Supplement 1).^32,33^

After adjustment for veteran- and facility-level covariables, veterans who received a low-value PSA test within the VHA underwent 31.2 (95% CI, 29.2 to 33.2) additional cascade services per 100 veterans compared with those who did not undergo low-value PSA testing. This included 12.3 (95% CI, 11.3 to 13.3) additional PSA tests per 100 veterans, 9.4 (95% CI, 8.4 to 10.5) additional related outpatient visits per 100 veterans, and 7.6 (95% CI, 6.6 to 8.7) additional urology visits per 100 veterans (Table 2). The total cost of potential cascade services among veterans who underwent PSA testing within the VHA was $1 277 453.5; compared with the control group, these veterans incurred an additional $24.5 (95% CI, $20.8 to $28.1) per veteran (Table 2).

Veterans who received a PSA test through Medicare underwent 39.3 (95% CI, 37.2 to 41.3) additional cascade services per 100 veterans compared with the control group. The most common potential cascade services were urology visits (16.6 additional services per 100 veterans [95% CI, 15.6 to 17.7]), additional PSA testing (13.3 additional services per 100 veterans [95% CI, 12.5 to 14.2]), and related outpatient visits (7.4 additional services per 100 veterans [95% CI, 6.5 to 8.3]) (Table 3). The total cost of potential cascade services among veterans who underwent PSA testing through Medicare was $811 602.2; compared with the control group, these veterans incurred an additional $35.9 (95% CI, $31.7 to $40.1) per veteran (Table 3).

Veterans who received low-value PSA testing through Medicare experienced 9.9 (95% CI, 9.7 to 10.1) additional cascade services per 100 veterans compared with those who underwent low-value PSA testing within the VHA, at an additional cost of $11.9 (95% CI, 7.6 to 16.2). This included 8.9 (95% CI, 8.8 to 9.0) additional urology visits and 2.6 (95% CI, 2.5 to 2.8) additional PSA tests. Veterans who underwent PSA testing within the VHA experienced 1.7 (95% CI, 1.70 to 1.72) additional related outpatient visits compared with those who underwent PSA testing through Medicare (Table 4).

In our sensitivity analysis that included veterans aged older than 83 years, we found that those who underwent PSA testing through Medicare experienced 5.6 (95% CI, 5.2 to 6.0) additional cascade services compared with those who underwent PSA testing within the VHA. The results were similar to our primary analyses when comparing the VHA PSA group vs the control group and the Medicare PSA group vs the control group (eTables 5-7 in Supplement 1).

We also examined the source of the potential cascade services. For veterans who underwent low-value PSA testing within the VHA, the unadjusted rate of total cascade services was 35.0 per 100 veterans within the VHA (15.8 for additional PSA testing, 18.9 for visits and diagnostic testing, and 0.4 for treatment) and 10.2 per 100 veterans within Medicare (2.9 for additional PSA testing, 7.0 for visits and diagnostic testing, and 0.3 for treatment). For veterans who underwent PSA testing within Medicare, the unadjusted rate of total cascade services was 13.8 per 100 veterans within the VHA (10.6 for additional PSA testing, 3.2 for visits and diagnostic testing, and <0.1 for treatment) and 40.1 per 100 veterans within Medicare (9.7 for additional PSA testing, 29.1 for visits and diagnostic testing, and 1.3 for treatment) (eTable 8 in Supplement 1).

## Discussion

In this cohort study of US veterans dually enrolled in VHA and Medicare, low-value PSA testing occurred in approximately 12.1% of those within the VHA and 5.9% of veterans through Medicare. Compared with veterans who did not undergo low-value PSA testing, those who underwent PSA testing within the VHA experienced an additional 31 potential cascade services per 100 veterans and those who underwent PSA testing through Medicare experienced an additional 39 potential cascade services per 100 veterans. When compared directly, veterans who underwent low-value PSA testing through Medicare experienced significantly more potential cascade services than those who underwent PSA testing within the VHA. The total cost of approximately $2 million associated with low-value care cascades was low compared with the $72.3 billion in total VHA expenditures in FY 2018. However, this does not account for indirect costs such as patient anxiety surrounding falsepositive results, inconvenience associated with downstream visits, and risks of invasive procedures.

This work is consistent with prior studies examining downstream implications of low-value PSA testing in older adults.^19-21^ For example, Zanwar et al^21^ compared downstream tests, treatments, and payments for prostate cancer care in older Texas Medicare beneficiaries cared for by primary care practitioners (PCPs) with high or low PSA screening rates. The authors similarly found that patients who were cared for by PCPs with high rates of PSA testing were more likely to visit a urologist or undergo prostate biopsy, ultrasound, or radiation compared with those who were cared for by PCPs with low rates of PSA testing. Like our study, Zanwar et al^21^ also found that payments for prostate cancer–related care were approximately $25 higher for those cared for by PCPs with high rates of PSA testing.

We build on prior studies by characterizing low-value PSA testing and subsequent care cascades in multiple health care systems. After adjusting for baseline patient and facility characteristics, we found that those who underwent initial PSA testing in Medicare experienced higher rates of care cascade services compared with those who underwent initial PSA testing within the VHA. We also found that approximately one-third of low-value PSA testing occurred outside of the VHA and that care cascade services often occurred in a different health care system than the initial PSA test. These findings are likely attributable to several factors such as differences in overutilization, access to care and cost to veterans, and duplicative efforts. For example, veterans who underwent initial testing in the Medicare system had higher rates of subsequent PSA tests, approximately half of which occurred in the VHA. This may represent VHA practitioners rechecking an abnormal result or the fact that they were unaware that a PSA test had occurred outside of the VHA. The higher rate of downstream care among veterans who underwent PSA testing in Medicare was also driven by higher rates of urology visits. This observation suggests that Medicare practitioners may be more likely to reflexively consult urology for abnormal PSA values, whereas VHA PCPs may continue to monitor on their own. Alternatively, this observation may suggest that veterans have greater access to urologists outside of the VHA compared to within it.

Our findings highlight both the difficulty and importance of better characterizing the often-fragmented care that veterans experience. To fully capture the extent to which veterans are subject to low-value care, we must examine not only downstream care stemming from an initial low-value service but also utilization occurring in multiple health care systems. This extends to other individuals seeking care in nonintegrated health care systems in which health information is not readily linked or shared. These findings have important clinical and policy implications. Although dual health system use has the potential to increase access to care, it is essential that health information be shared between VHA and non-VHA practitioners and more broadly, across health systems in general. Additionally, VHA interventions to reduce low-value care that focus solely on VHA practitioners are likely to have attenuated effects, and broader interventions focused on dual use in general may be warranted.

### Limitations

Our study has several important limitations. Using a claims-based approach lacks clinical granularity. In using broad exclusion criteria to establish our cohort and applying an age cutoff of 75 rather than 70 years to identify low-value PSA testing, we hoped to better capture truly low-value testing but acknowledge that we may have excluded some veterans, such as those with mildly elevated PSA, for whom PSA testing would be considered low value. Similarly, we were unable to definitively identify whether cascade services occurred as a result of the initial PSA test. By using a comparison group, similar to prior studies examining care cascades, we attempted to account for the baseline level of care occurring in our study sample.^11^ We also chose an outcome period of 6 months so as to not overcapture outcomes not attributable to the initial PSA test. In doing so, we may have failed to capture services such as surgical procedures or differences in cascade services over time as practitioners deintensify watchful waiting and/or active surveillance. Additionally, the VHA services identified in this study only included those delivered through the VHA and not through VA Community Care (VACC). However, prior work has shown that rates of low-value PSA testing delivered through VACC are low.^17^ Lastly, cost data were unavailable for surgical procedures; however, these occurred at very low rates within our study and likely contributed little to the overall additional costs.

## Conclusions

The findings of this cohort study suggest that low-value PSA testing and subsequent care cascades were commonly experienced among US veterans dually enrolled in the VHA and Medicare. A substantial proportion of the initial low-value tests and downstream care occurred in non-VHA health care settings through Medicare, demonstrating that we must examine care across multiple health care systems to fully understand and ultimately develop policies to reduce low-value health service use among VHA-enrolled veterans.

## Supplementary Material

Online only supplement **eTable 1.** Cohort Inclusion and Exclusion Criteria, Index Low-Value Prostate-Specific Antigen Testing, and Potential Cascade Services With Relevant Administrative Codes
**eMethods.** Generation of Inverse Probability of Treatment Weights
**eTable 2.** Standardized Mean Differences for Baseline Patient- and Facility-Level Characteristics Between Veterans Who Underwent Low-Value Prostate-Specific Antigen Testing Within the US Veterans Health Administration and Those Who Did Not Undergo Low-Value PSA Testing Before and After Inverse Probability of Treatment Weighting
**eTable 3.** Standardized Mean Differences for Baseline Patient- and Facility-Level Characteristics Between Veterans Who Underwent Low-Value Prostate-Specific Antigen Testing Within Medicare and Those Who Did Not Undergo Low-Value PSA Testing Before and After Inverse Probability of Treatment Weighting
**eTable 4.** Standardized Mean Differences for Baseline Patient- and Facility-Level Characteristics Between Veterans Who Underwent Low-Value Prostate-Specific Antigen Testing Within the US Veterans Health Administration and Medicare Before and After Inverse Probability of Treatment Weighting
**eTable 5.** Difference in Use of Potential Cascade Services in Veterans Aged Older than 83 Years Who Received Low-Value Prostate-Specific Antigen Testing Within the US Veterans Health Administration Versus Those Who Did Not Receive Low-Value PSA Testing
**eTable 6.** Difference in Use of Potential Cascade Services in Veterans Aged Older than 83 Years Who Received Low-Value Prostate-Specific Antigen Testing Within Medicare Versus Those Who Did Not Receive Low-Value PSA Testing
**eTable 7.** Difference in Use of Potential Cascade Services in Veterans Aged Older than 83 Years Who Received Low-Value Prostate-Specific Antigen Testing Within Medicare Versus Those Who Received Low-Value PSA Testing Within the US Veterans Health Administration
**eTable 8.** Rates of Potential Cascade Services After Low-Value Prostate-Specific Antigen Testing by Health Care System

## Figures and Tables

**Figure.**
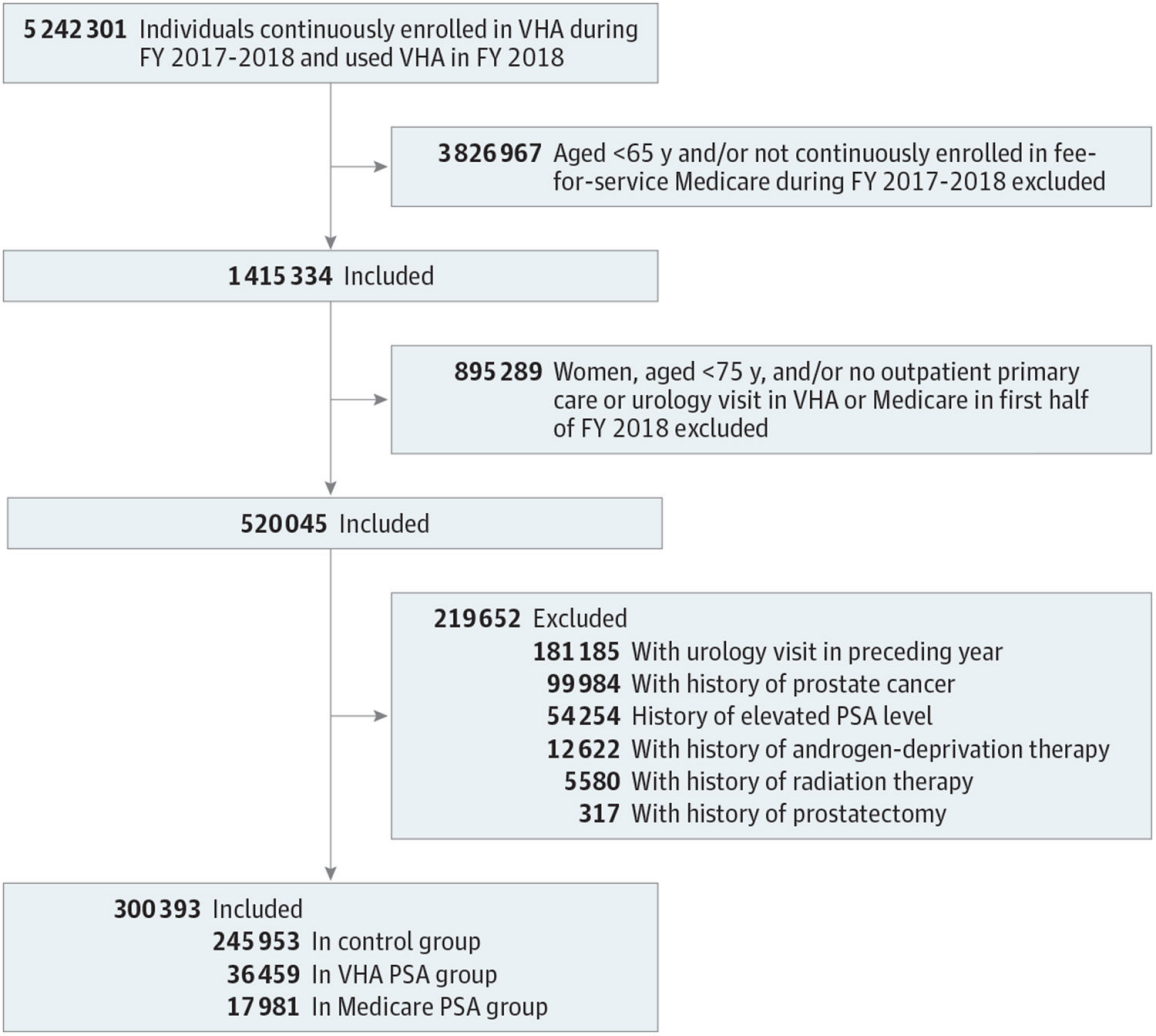
Study FlowDiagram Veterans Health Administration (VHA) and Medicare fee-for-service dual enrollees (N = 300 393) at risk of receiving a low-value prostate-specific antigen (PSA) test in fiscal year (FY) 2018. Exclusion criteria are not mutually exclusive.

**Table 1.** Baseline Patient- and Facility-Level Characteristics of US Veterans Who Underwent Low-Value Prostate-Specific Antigen (PSA) Testing in the First Half of Fiscal Year 2018 vs Those Who Did Not^a^

Characteristic	Final cohort (N = 300 393)	VHA PSA group (n = 36 459)^b^	Medicare PSA group (n = 17 981)^b^	Control group (n = 245 953)^b^
Patient level				
Age, mean (SD), y	82.6 (5.6)	80.0 (4.7)	81.3 (5.2)	83.1 (5.6)
Race and ethnicity, No. (%)				
Hispanic	8760 (2.9)	1170 (3.2)	464 (2.6)	7126 (2.9)
Non-Hispanic Black	20 134 (6.7)	2653 (7.3)	1055 (5.9)	16 426 (6.7)
Non-Hispanic White	264 411 (88.0)	31 824 (87.3)	16 039 (89.2)	216 548 (88.0)
Other non-Hispanic racial or ethnic minority group or multiracial^c^	7088 (2.4)	812 (2.2)	423 (2.4)	5853 (2.4)
VA priority group, No. (%)^d^				
1-4	119 194 (39.7)	14 978 (41.1)	7528 (41.9)	96 688 (39.3)
5	67 314 (22.4)	8369 (23.0)	2355 (13.1)	56 590 (23.0)
6-8	113 885 (37.9)	13 112 (36.0)	8098 (45.0)	92 675 (37.7)
Driving distance to the nearest VA facility, mean (SD), miles	17.1 (16.9)	18.8 (18.8)	16.6 (15.8)	16.8 (16.6)
No. of Elixhauser conditions, mean (SD)^e^	2.8 (2.3)	2.3 (2.1)	3.0 (2.2)	2.8 (2.3)
Facility level				
Academic affiliation, yes, No. (%)	293 929 (97.9)	35 663 (97.8)	17 719 (98.5)	240 547 (97.8)
Facility size, total No. outpatient visits fiscal year 2018, mean (SD)	745 068 (402 244)	755 774 (437 479)	788 769 (419 935)	740 286 (395 187)
Census region, No. (%)				
Northeast	47 613 (15.9)	3502 (9.6)	2917 (16.2)	41 194 (16.8)
Midwest	73 782 (24.6)	8402 (23.1)	3812 (21.2)	61 568 (25.0)
South	122 801 (40.9)	17 752 (48.7)	8505 (47.3)	96 544 (39.3)
West	56 197 (18.7)	6803 (18.7)	2747 (15.3)	46 647 (19.0)
Rurality, No. (%)				
Large metropolitan	101 455 (33.8)	9938 (27.3)	6685 (37.3)	84 832 (34.5)
Small metropolitan	114 668 (38.2)	14 582 (40.0)	7031 (39.2)	93 055 (37.9)
Micropolitan	46 668 (15.6)	6222 (17.1)	2441 (13.6)	38 005 (15.5)
Noncore rural	37 189 (12.4)	5687 (15.6)	1789 (10.0)	29 713 (12.1)
Facility complexity level, No. (%)^f^				
High	229 022 (76.2)	26 709 (73.3)	14 161 (78.8)	188 152 (76.5)
Medium	35 831 (11.9)	4587 (12.6)	1859 (10.3)	29 385 (12.0)
Low	35 540 (11.8)	5163 (14.2)	1961 (10.9)	28 416 (11.6)

Abbreviations: VA, US Department of Veterans Affairs; VHA, US Veterans Health Administration.

a Values are presented before applying inverse probability of treatment weighting. Missing values for race and ethnicity, VA priority group, driving distance to the nearest VA facility, census region, and rurality were generated using single imputation.

b All groups had statistically significant differences in characteristics by analysis of variance for continuous variables and χ^2^ for categorical variables (*P* < .05).

c Other non-Hispanic racial or ethnic minority group includes American Indian or Alaska Native, Asian, and Native Hawaiian or other Pacific Islander.

d Veterans are assigned to 1 of 8 priority groups at VA enrollment based on service-connected illnesses, era of service, and socioeconomic status determined by means testing. Priority group determines level of copayment. Priority groups are condensed for presentation here based on similarity of copays between groups but were included separately in our models.

e Individual Elixhauser conditions are shown in eTables 2 to 4 in Supplement 1.

f The complexity rating is based on a VA medical center’s patient volume, number and breadth of physician specialists, patient case mix, intensive care unit capabilities, and degree of teaching and research.

**Table 2.** Use and Cost of Potential Cascade Services Among US Veterans Who Received Low-Value Prostate-Specific Antigen (PSA) Testing Within the US Veterans Health Administration (VHA) vs Those Who Did Not Receive Low-Value PSA Testing^a^

	Utilization rate per 100 veterans				Cost per veteran, US$			
	Unadjusted				Mean unadjusted			
Cascade service	VHA PSA group (n = 36 459)	Control group (n = 245 953)	Unadjusted difference in rate^b^	Adjusted difference in rate (95% CI)^c^	VHA PSA group (n = 36 459)	Control group (n = 245 953)	Unadjusted difference^d^	Adjusted difference (95% CI)^c^
Total	45.3	14.4	30.9	31.2 (29.2 to 33.2)	35.0	9.8	25.2	24.5 (20.8 to 28.1)
Additional PSA test	18.6	5.2	13.4	12.3 (11.3 to 13.3)	4.2	1.2	3.0	2.8 (2.5 to 3.1)
Related outpatient visit^e^	10.0	1.4	8.6	9.4 (8.4 to 10.5)^f^	7.5	1.1	6.4	7.1 (6.1 to 8.1)
Urology visit	14.2	7.3	6.9	7.6 (6.6 to 8.7)	10.2	5.4	4.8	5.2 (4.1 to 6.3)
Prostate imaging	1.3	0.5	0.8	0.8 (0.7 to 1.0)	4.1	1.3	2.8	2.9 (2.0 to 3.8)
Prostate biopsy	0.42	0.03	0.40	0.3 (0.2 to 0.4)	7.8	0.5	7.3	5.6 (4.4 to 6.7)
Androgen deprivation therapy	0.38	0.03	0.35	0.5 (0.3 to 0.6)	0.9	0.4	0.5	0.6 (0.3 to 0.9)
Surgical procedure^g^	<0.1^h^	<0.1^h^	<0.1^h^	<0.1^h^	NA	NA	NA	NA
Radiation therapy^i^	0.33	0.04	0.29	0.3 (0.1 to 0.4)	0.33	0.06	0.27	0.3 (−0.2 to 0.8)

Abbreviation: NA, not applicable.

a Outcomes identified during the 6-month follow-up period.

b Determined by subtracting the rate of potential cascade services per 100 veterans among those who did not receive low-value PSA testing from those who received low-value PSA testing within the VHA.

c Adjusted for patient-level and US Department of Veterans Affairs (VA) medical center–level covariates (age, race and ethnicity, VA priority group, driving distance to the nearest VA facility, number of Elixhauser conditions, individual Elixhauser conditions, academic affiliation, facility size, census region, rurality, and complexity level) using inverse probability of treatment weighting.

d Determined by subtracting the cost of potential cascade services per veteran among those who did not receive low-value PSA testing from those who received low-value PSA testing within the VHA.

e Outpatient visit or consultation with a primary diagnosis of prostate cancer or elevated PSA.

f Consists of 4.0 (95% CI, 3.5 to 4.5) visits for elevated PSA and 5.5 (95% CI, 4.7 to 6.2) visits for prostate cancer.

g Prostatectomy or surgical resection of the prostate. Cost data not available for surgical procedures.

h Cell size suppressed due to Medicare data use agreements.

i Contact radiation, beam radiation, insertion of radioactive element, brachytherapy, intraoperative radiation therapy, or plaque radiation of the prostate.

**Table 3.** Use and Cost of Potential Cascade Services Among US Veterans Who Received Low-Value Prostate-Specific Antigen (PSA) Testing Within Medicare vs Those Who Did Not Receive Low-Value PSA Testing^a^

	Utilization rate per 100 veterans				Cost per veteran, US$			
	Unadjusted				Mean unadjusted			
Cascade service	Medicare PSA group (n = 17 981)	Control group (n = 245 953)	Unadjusted difference^b^	Adjusted difference (95% CI)^c^	Medicare PSA group (n = 17 981)	Control group (n = 245 953)	Unadjusted difference^d^	Adjusted difference (95% CI)^c^
Total	53.9	14.4	39.5	39.3 (37.2 to 41.3)	45.1	9.8	35.3	35.9 (31.7 to 40.1)
Additional PSA test	20.3	5.2	15.1	13.3 (12.5 to 14.2)	4.5	1.2	3.4	3.0 (2.7 to 3.2)
Related outpatient visit^e^	8.1	1.4	6.7	7.4 (6.5 to 8.3)^f^	6.4	1.1	5.3	5.8 (5.1 to 6.6)
Urology visit	23.2	7.3	15.9	16.6 (15.6 to 17.7)	18.2	5.4	12.9	13.4 (12.4 to 14.4)
Prostate imaging	0.3	0.5	−0.2	−0.11 (−0.14 to −0.08)	1.0	1.3	−0.3	−0.2 (−0.5 to 0.1)
Prostate biopsy	0.73	0.03	0.70	0.7 (0.5 to 0.8)	13.3	0.5	12.9	12.4 (9.4 to 15.5)
Androgen deprivation therapy	0.53	0.03	0.50	0.6 (0.4 to 0.9)	1.2	0.4	0.8	1.0 (0.6 to 1.4)
Surgical procedure^g^	<0.1^h^	<0.1^h^	<0.1^h^	<0.1^h^	NA	NA	NA	NA
Radiation therapy^i^	0.81	0.04	0.77	0.6 (0.4 to 0.9)	0.50	0.06	0.45	0.4 (0.1 to 0.7)

Abbreviation: NA, not applicable.

a Outcomes identified during 6-month follow-up period.

b Determined by subtracting the rate of potential cascade services per 100 veterans in those who did not receive low-value PSA testing from those who received low-value PSA testing within Medicare.

c Adjusted for patient-level and US Department of Veterans Affairs (VA) medical center–level covariates (age, race and ethnicity, VA priority group, driving distance to the nearest VA facility, number of Elixhauser conditions, individual Elixhauser conditions, academic affiliation, facility size, census region, rurality, complexity level) using inverse probability of treatment weighting.

d Determined by subtracting the cost of potential cascade services per veteran among those who did not receive low-value PSA testing from those who received low-value PSA testing within Medicare.

e Outpatient visit or consultation with a primary diagnosis of prostate cancer or elevated PSA.

f Consists of 4.8 (95% CI, 4.3 to 5.3) visits for elevated PSA and 2.5 (95% CI, 2.0 to 3.1) visits for prostate cancer.

g Prostatectomy or surgical resection of the prostate. Cost data not available for surgical procedures.

h Cell size suppressed due to Medicare data use agreements.

i Contact radiation, beam radiation, insertion of radioactive element, brachytherapy, intraoperative radiation therapy, or plaque radiation of the prostate.

**Table 4.** Use and Cost of Potential Cascade Services Among US Veterans Who Received Low-Value Prostate-Specific Antigen (PSA) Testing Within the US Veterans Health Administration (VHA) vs Medicare^a^

	Utilization rate per 100 veterans				Cost per veteran, US$			
	Unadjusted				Mean unadjusted			
Cascade services	Medicare PSA group (n = 17 981)	VHA PSA group (n = 36 459)	Unadjusted difference^b^	Adjusted difference (95% CI)^c^	Medicare PSA group (n = 17 981)	VHA PSA group (n = 36 459)	Unadjusted difference^d^	Adjusted difference (95% CI)^c^
Total	53.9	45.3	8.6	9.9 (9.7 to 10.1)	45.1	35.0	10.1	11.9 (7.6 to 16.2)
Additional PSA test	20.3	18.6	1.7	2.6 (2.5 to 2.8)	4.5	4.2	0.3	0.6 (0.5 to 0.6)
Related outpatient visit^e^	8.1	10.0	−1.9	−1.7 (−1.72 to −1.70)^f^	6.4	7.5	−1.1	−1.0 (−1.8 to −0.2)
Urology visit	23.2	14.2	9.0	8.9 (8.8 to 9.0)	18.2	10.2	8.0	8.0 (6.6 to 9.4)
Prostate imaging	0.3	1.3	−1.0	−0.9 (−0.8 to −1.0)	1.0	4.1	−3.1	−3.0 (−3.6 to −2.3)
Prostate biopsy	0.73	0.42	0.31	0.4 (0.3 to 0.5)	13.3	7.8	5.5	6.8 (4.1 to 9.5)
Androgen deprivation therapy	0.53	0.38	0.15	0.12 (0.06 to 0.19)	1.2	0.9	0.3	0.3 (−0.1 to 0.7)
Surgical procedure^g^	<0.1^h^	<0.1^h^	<0.1^h^	<0.1^h^	NA	NA	NA	NA
Radiation therapy^i^	0.81	0.33	0.48	0.47 (0.27 to 0.68)	0.50	0.33	NA	0.6 (0.2 to 0.9)

Abbreviation: NA, not applicble.

a Outcomes identified during 6-month follow-up period.

b Determined by subtracting the rate of potential cascade services per 100 veterans among those who received low-value PSA testing within the VHA from those who received low-value PSA testing within Medicare.

c Adjusted for patient-level and US Department of Veterans Affairs (VA) medical center–level covariates (age, race and ethnicity, VA priority group, driving distance to the nearest VA facility, number of Elixhauser conditions, individual Elixhauser conditions, academic affiliation, facility size, census region, rurality, and complexity level) using inverse probability of treatment weighting.

d Determined by subtracting the cost of potential cascade services per veteran among those who received low-value PSA testing within the VHA from those who received low-value PSA testing within Medicare.

e Outpatient visit or consultation with a primary diagnosis of prostate cancer or elevated PSA.

f Consists of 0.8 (95% CI, 0.7 to 1.0) visits for elevated PSA and −2.5 (95% CI, −2.47 to −2.63) visits for prostate cancer.

g Prostatectomy or surgical resection of the prostate. Cost data not available for surgical procedures.

h Cell size suppressed due to Medicare data use agreements.

i Contact radiation, beam radiation, insertion of radioactive element, brachytherapy, intraoperative radiation therapy, or plaque radiation of the prostate.

## Data Availability

See Supplement 2.

## Key Points

### Question

What is the frequency, cost, and source of low-value prostate-specific antigen (PSA) testing and subsequent care cascades among veterans dually enrolled in the US Veterans Health Administration (VHA) and Medicare systems?

### Findings

In this cohort study of 300 393 dually enrolled US male veterans, those who underwent low-value PSA testing within the VHA vs Medicare experienced 31 vs 39 additional cascade services per 100 veterans, respectively, compared with the control group. Veterans who underwent low-value PSA testing through Medicare experienced substantially more cascade services (9.9 additional cascade services per 100 veterans) than those who underwent PSA testing within the VHA.

### Meaning

Care cascades after low-value PSA testing are common among veterans dually enrolled in the VHA and Medicare and occur through multiple sources of care.
