# Supplementary material for: Assessment of Care Cascades Following Low-Value Prostate-Specific Antigen Testing Among Veterans Dually Enrolled in the US Veterans Health Administration and Medicare Systems
Source: JAMA Netw Open. Author manuscript; Available in PMC 2023 Jan 30. (PMC9856440; doi:10.1001/jamanetworkopen.2022.47180)
Supplement: Online only supplement — eTable 1. Cohort Inclusion and Exclusion Criteria, Index Low-Value Prostate-Specific Antigen Testing, and Potential Cascade Services With Relevant Administrative Codes eMethods. Generation of Inverse Probability of Treatment Weights eTable 2. Standardized Mean Differences for Baseline Patient- and Facility-Level Characteristics Between Veterans Who Underwent Low-Value Prostate-Specific Antigen Testing Within the US Veterans Health Administration and Those Who Did Not Undergo Low-Value PSA Testing Before and After Inverse Probability of Treatment Weighting eTable 3. Standardized Mean Differences for Baseline Patient- and Facility-Level Characteristics Between Veterans Who Underwent Low-Value Prostate-Specific Antigen Testing Within Medicare and Those Who Did Not Undergo Low-Value PSA Testing Before and After Inverse Probability of Treatment Weighting eTable 4. Standardized Mean Differences for Baseline Patient- and Facility-Level Characteristics Between Veterans Who Underwent Low-Value Prostate-Specific Antigen Testing Within the US Veterans Health Administration and Medicare Before and After Inverse Probability of Treatment Weighting eTable 5. Difference in Use of Potential Cascade Services in Veterans Aged Older than 83 Years Who Received Low-Value Prostate-Specific Antigen Testing Within the US Veterans Health Administration Versus Those Who Did Not Receive Low-Value PSA Testing eTable 6. Difference in Use of Potential Cascade Services in Veterans Aged Older than 83 Years Who Received Low-Value Prostate-Specific Antigen Testing Within Medicare Versus Those Who Did Not Receive Low-Value PSA Testing eTable 7. Difference in Use of Potential Cascade Services in Veterans Aged Older than 83 Years Who Received Low-Value Prostate-Specific Antigen Testing Within Medicare Versus Those Who Received Low-Value PSA Testing Within the US Veterans Health Administration eTable 8. Rates of Potential Cascade Services After Low-Value Prostate-Specific Antigen Testing by Health Care S [file NIHMS1866733-supplement-Online_only_supplement.pdf]

Supplementary Online Content

Pickering AN, Zhao X, Sileanu FE, et al. Assessment of care cascades following low-value prostate-specific antigen testing among veterans dually enrolled in the US Veterans Health Administration and Medicare systems. *JAMA Netw Open*. 2022;5(12):e2247180. doi:10.1001/jamanetworkopen.2022.47180

eTable 1. Cohort Inclusion and Exclusion Criteria, Index Low-Value Prostate-Specific Antigen Testing, and Potential Cascade Services With Relevant Administrative Codes
eMethods. Generation of Inverse Probability of Treatment Weights
eTable 2. Standardized Mean Differences for Baseline Patient- and Facility-Level Characteristics Between Veterans Who Underwent Low-Value Prostate-Specific Antigen Testing Within the US Veterans Health Administration and Those Who Did Not Undergo Low-Value PSA Testing Before and After Inverse Probability of Treatment Weighting
eTable 3. Standardized Mean Differences for Baseline Patient- and Facility-Level Characteristics Between Veterans Who Underwent Low-Value Prostate-Specific Antigen Testing Within Medicare and Those Who Did Not Undergo Low-Value PSA Testing Before and After Inverse Probability of Treatment Weighting
eTable 4. Standardized Mean Differences for Baseline Patient- and Facility-Level Characteristics Between Veterans Who Underwent Low-Value Prostate-Specific Antigen Testing Within the US Veterans Health Administration and Medicare Before and After Inverse Probability of Treatment Weighting
eTable 5. Difference in Use of Potential Cascade Services in Veterans Aged Older than 83 Years Who Received Low-Value Prostate-Specific Antigen Testing Within the US Veterans Health Administration Versus Those Who Did Not Receive Low-Value PSA Testing
eTable 6. Difference in Use of Potential Cascade Services in Veterans Aged Older than 83 Years Who Received Low-Value Prostate-Specific Antigen Testing Within Medicare Versus Those Who Did Not Receive Low-Value PSA Testing
eTable 7. Difference in Use of Potential Cascade Services in Veterans Aged Older than 83 Years Who Received Low-Value Prostate-Specific Antigen Testing Within Medicare Versus Those Who Received Low-Value PSA Testing Within the US Veterans Health Administration
eTable 8. Rates of Potential Cascade Services After Low-Value Prostate-Specific Antigen Testing by Health Care System

This supplementary material has been provided by the authors to give readers additional information about their work.

© 2022 Pickering AN et al. *JAMA Network Open*

eTable 1. Cohort Inclusion and Exclusion Criteria, Index Low-Value Prostate-Specific Antigen Testing, and Potential Cascade Services With Relevant Administrative Codes

**Inclusion criteria**	**Exclusion criteria**	**Index low-value PSA test**	**Potential cascade services**
(1) Male<br>(2) Aged ≥ 75<br>(3) At least one outpatient primary care or urology visit in VHA or Medicare in the first half of FY18	History of the following within one year prior to the date of the index PSA test or index outpatient visit:<br><br>(1) Prostate cancer<br>ICD-10: C61, Z85.46, D29.1, D07.5, D40.0<br><br>(2) Elevated PSA test<br>ICD-10: R97.20, R97.21<br><br>(3) Prostatectomy<br>CPT: 725801, 55810, 55812, 55815, 55840, 55842, 55845, 55866<br><br>(4) Radiation therapy<br>CPT: 77401-2, 77407, 77412, 77417, 77423, 77424, 77425, 77427, 77431, 77432, 77435, 77469, 77470, 77499, 77750, 77761, 77762, 77763, 77767, 77768, 77770, 77771, 77772, 77778, 77789, 77790, 77799<br><br>(5) Androgen deprivation therapy<br>CPT: 54520, 54522 (surgical)<br>HCPCS: J1950, J9217, J9218, J9219, J3315, J9202 (medical)<br><br>(4) Urology visit<br>Stop code (VHA data): 414<br>Physician specialty code (Medicare data): 34	CPT: G1012-3, 84152-3	PSA test<br><br>CPT: G1012-3, 84152-3
			Outpatient visit or consultation for elevated PSA or prostate cancer

© 2022 Pickering AN et al. *JAMA Network Open*

			CPT: 99201-5, 99211-5 (outpatient visit or consultation)<br><br>Associated with primary diagnosis of<br>ICD-10: C61, Z85.46 (malignant neoplasm of prostate)<br>D29.1 (benign neoplasm of prostate)<br>D07.5 (carcinoma in situ of prostate)<br>R97.20-1 (elevated PSA)<br><br>*Excluding those visits associated with administrative codes for urology visit
			Outpatient visit to urologist<br><br>CPT: 99201-5, 99211-5 (outpatient visit or consultation)<br><br>Associated with Stop code: 414 (urology) for VHA data or Physician specialty code: 34 for Medicare data
			Prostate imaging<br><br>CPT: 76872-3 (Transrectal US)<br>72192-4 (CT pelvis)<br>72195-7 (MRI pelvis)<br>78300-78305, 78306, 78315, 78350-1, 78399 (bone scan)<br><br>*No current CPT codes for prostate MRI
			Prostate biopsy<br><br>CPT: 55700, 55705, 55706<br><br>HCPCS: G0416

© 2022 Pickering AN et al. *JAMA Network Open*

			Androgen deprivation therapy<br><br><u>CPT</u>: 54520, 54522 (surgical)<br><br><u>HCPCS</u>: J1950, J9217, J9218, J9219, J3315, J9202 (medical)
			Surgical procedure<br><br><u>CPT</u>: 55801 (excision procedures on prostate)<br>55810, 55812, 55815, 55821, 55831, 55840, 55842, 55845 (prostatectomy)<br>55866 (laparoscopic procedures on the prostate)
			Radiation therapy<br><br><u>CPT</u>: 77401-2, 77407, 77412, 77417, 77423, 77424, 77425, 77427, 77431, 77432, 77435, 77469, 77470, 77499, 77750, 77761, 77762, 77763, 77767, 77768, 77770, 77771, 77772, 77778, 77789, 77790, 77799 (radiation therapy)<br><br>With primary diagnosis of prostate cancer during the outcome period

© 2022 Pickering AN et al. *JAMA Network Open*

eMethods. Generation of Inverse Probability of Treatment Weights

To generate the stabilized IPTWs, we created three separate multivariable logistic regression models to estimate the predicted probability of each Veteran having received their respective "treatments" (i.e., PSA testing within VHA or no PSA testing, PSA testing within Medicare or no PSA testing, PSA testing within VHA or PSA testing with Medicare) based on the observed covariates and converted these probabilities to stabilized IPTWs. We then applied the weights to the negative binomial models and calculated the difference in adjusted rates of each individual cascade service between the VHA PSA group and the control group, the Medicare PSA group and the control group and the VHA PSA group and the Medicare PSA group.

© 2022 Pickering AN et al. *JAMA Network Open*

eTable 2. Standardized Mean Differences for Baseline Patient- and Facility-Level Characteristics Between Veterans Who Underwent Low-Value Prostate-Specific Antigen Testing Within the US Veterans Health Administration and Those Who Did Not Undergo Low-Value PSA Testing Before and After Inverse Probability of Treatment Weighting[a]

	**Unweighted**			**Weighted[b]**		
	**VHA PSA Group (n=36,459)**	**Control Group (n=245,953)**	**SMD[c]**	**VHA PSA Group (n=36,459)**	**Control Group (n=245,953)**	**SMD[c]**
**Patient-level characteristics**						
Age, y, mean (SD)	80.0 (4.7)	83.1 (5.6)	0.60	83.0 (6.0)	82.7 (5.6)	0.06
Race/ethnicity, n (%)						
Hispanic	1170 (3.2)	7126 (2.9)	0.02	1055.2 (2.9)	7218.7 (2.9)	0.00
Non-Hispanic black	2653 (7.3)	16426 (6.7)	0.02	2571.9 (7.1)	16627.7 (6.8)	0.01
Non-Hispanic other + Multiracial	812 (2.2)	5853 (2.4)	0.01	827.6 (2.3)	5802.1 (2.4)	0.01
Non-Hispanic white	31824 (87.3)	216548 (88.0)	ref	32,004.3 (87.8)	216304.5 (88.0)	ref
VA priority group[d], n (%)						
1	7716 (21.2)	46470 (18.9)	ref	7051.6 (19.3)	47209.0 (19.2)	ref
2	2247 (6.2)	14590 (5.9)	0.03	2101.7 (5.8)	14656.9 (6.0)	0.02
3	4389 (12.0)	27851 (11.3)	0.03	4126.2 (11.3)	28076.7 (11.4)	0.01
4	626 (1.7)	7777 (3.2)	0.22	1171.6 (3.2)	7322.6 (3.0)	0.02
5	8369 (23.0)	56590 (23.0)	0.06	8413.6 (23.1)	56584.0 (23.0)	0.00
6	866 (2.4)	3754 (1.5)	0.09	576.3 (1.6)	4019.6 (1.6)	0.01
7	1155 (3.2)	10588 (4.3)	0.15	1602.9 (4.4)	10231.5 (4.2)	0.02
8	11091 (30.4)	78333 (31.9)	0.08	11415.0 (31.3)	77852.6 (31.7)	0.01
Driving distance to nearest VA facility, miles, mean (SD)	18.8 (18.8)	16.8 (16.6)	0.11	16.8 (17.2)	17.1 (16.9)	0.02
Number of Elixhauser conditions, mean (SD)	2.3 (2.1)	2.8 (2.3)	0.24	2.8 (2.4)	2.8 (2.3)	0.02
HIV/AIDS, n (%)	37 (0.1)	207 (0.1)	0.01	33.1 (0.1)	213.3 (0.1)	0.00
Alcohol use disorder	446 (1.2)	3256 (1.3)	0.01	456.1 (1.3)	3224.4 (1.3)	0.01
Iron-deficiency anemia	4092 (11.2)	40747 (16.6)	0.15	6081.9 (16.7)	39073.2 (15.9)	0.02
Rheumatoid/collagen-vascular disease	1018 (2.8)	7496 (3.1)	0.02	1085.2 (3.0)	7413.1 (3.0)	0.00

© 2022 Pickering AN et al. *JAMA Network Open*

	Unweighted			Weighted[b]		
	VHA PSA Group (n=36,459)	Control Group (n=245,953)	SMD[c]	VHA PSA Group (n=36,459)	Control Group (n=245,953)	SMD[c]
**Patient-level characteristics**						
Blood loss anemia	240 (0.7)	2557 (1.0)	0.04	398.2 (1.1)	2437.7 (1.0)	0.01
Congestive heart failure	3602 (9.9)	37165 (15.1)	0.16	5431.7 (14.9)	35519.2 (14.4)	0.01
Chronic pulmonary disease	6312 (17.3)	49576 (20.2)	0.07	7319.0 (20.1)	48688.9 (19.8)	0.01
Coagulation deficiency	928 (2.6)	9298 (3.8)	0.07	1313.9 (3.6)	8907.6 (3.6)	0.00
Depression	2665 (7.3)	20650 (8.4)	0.04	3014.5 (8.3)	20307.0 (8.3)	0.00
Diabetes without chronic conditions	4867 (13.4)	31515 (12.8)	0.02	4783.8 (13.1)	31698.1 (12.9)	0.01
Diabetes with chronic conditions	7064 (19.4)	55320 (22.5)	0.08	8103.0 (22.2)	54334.7 (22.1)	0.00
Drug abuse	151 (0.4)	838 (0.3)	0.01	127.8 (0.4)	862.1 (0.4)	0.00
Uncomplicated hypertension	18699 (51.3)	132740 (54.0)	0.05	19586.0 (53.7)	131899.8 (53.6)	0.00
Complicated hypertension	4589 (12.6)	43349 (17.6)	0.14	6398.9 (17.6)	41769.3 (17.0)	0.02
Hypothyroidism	3836 (10.5)	32124 (13.1)	0.08	4790.2 (13.1)	31334.5 (12.7)	0.01
Liver disease	516 (1.4)	3824 (1.6)	0.01	548.3 (1.5)	3781.1 (1.5)	0.00
Lymphoma	389 (1.1)	2982 (1.2)	0.01	481.3 (1.3)	2940.2 (1.2)	0.01
Fluid and electrolyte disorder	2416 (6.6)	23796 (9.7)	0.11	3453.5 (9.5)	22839.5 (9.3)	0.01
Metastatic cancer	150 (0.4)	1454 (0.6)	0.03	251.1 (0.7)	1398.7 (0.6)	0.02
Other neurologic disorder	2842 (7.8)	28685 (11.7)	0.13	4096.2 (11.2)	27458.1 (11.2)	0.00
Obesity	3147 (8.6)	21019 (8.6)	0.00	3107.6 (8.5)	21047.9 (8.6)	0.00
Paralysis	491 (1.4)	4420 (1.8)	0.04	624.0 (1.7)	4278.1 (1.7)	0.00
Peripheral vascular disease	4327 (11.9)	39846 (16.2)	0.12	5902.7 (16.2)	38482.4 (15.7)	0.01
Psychosis	600 (1.7)	4659 (1.9)	0.02	704.2 (1.9)	4581.6 (1.9)	0.01
Pulmonary circulation disorder	415 (1.1)	3594 (1.5)	0.03	540.4 (1.5)	3493.1 (1.4)	0.01
Renal failure	4586 (12.6)	46073 (18.7)	0.17	6814.3 (18.7)	44140.5 (18.0)	0.02
Solid tumor without metastasis	1140 (3.1)	8702 (3.5)	0.02	1306.3 (3.6)	8571.1 (3.5)	0.01

© 2022 Pickering AN et al. *JAMA Network Open*

	Unweighted			Weighted[b]		
	VHA PSA Group (n=36,459)	Control Group (n=245,953)	SMD[c]	VHA PSA Group (n=36,459)	Control Group (n=245,953)	SMD[c]
**Patient-level characteristics**						
Peptic ulcer disease	224 (0.6)	1978 (0.8)	0.02	286.6 (0.8)	1916.1 (0.8)	0.00
Valvular disease	2910 (8.0)	27728 (11.3)	0.11	4054.1 (11.1)	26688.1 (10.9)	0.01
Weight loss	688 (1.9)	7586 (3.1)	0.08	1170.6 (3.2)	7209.3 (2.9)	0.02
**Facility-level characteristics**						
Academic affiliation, yes, n (%)	35663 (97.8)	240547 (97.8)	0.00	35659.8 (97.8)	240548.3 (97.8)	0.00
Facility size, total # outpatient visits FY18, mean (SD)	755774 (437479)	740286 (395187)	0.04	742368.9 (408548.3)	742218 (398369.6)	0.00
Census region, n (%)						
Northeast	3502 (9.6)	41194 (16.8)	ref	6105.3 (16.8)	38948.3 (15.8)	ref
Midwest	8402 (23.1)	61568 (25.0)	0.23	8771.6 (24.1)	60915.4 (24.8)	0.04
South	17752 (48.7)	96544 (39.3)	0.32	14677.1 (40.3)	99543.3 (40.5)	0.03
West	6803 (18.7)	46647 (19.0)	0.27	6905.0 (18.9)	46546.0 (18.9)	0.03
Rurality, n (%)						
Large metropolitan	9938 (27.3)	84832 (34.5)	ref	12543.7 (34.4)	82642.3 (33.6)	ref
Small metropolitan	14582 (40.0)	93055 (37.9)	0.14	13782.6 (37.8)	93897.0 (38.2)	0.02
Micropolitan	6222 (17.1)	38005 (15.5)	0.16	5652.4 (15.5)	38560.6 (15.7)	0.02
Noncore rural	5687 (15.6)	29713 (12.1)	0.23	4480.4 (12.3)	30853.1 (12.5)	0.02
Facility complexity level[e],n (%)						
High complexity	26709 (73.3)	188152 (76.5)	ref	28163.9 (77.3)	187173.3 (76.1)	ref
Medium complexity	4587 (12.6)	29385 (12.0)	0.03	4122.0 (11.3)	29555.2 (12.0)	0.03
Low complexity	5163 (14.2)	28416 (11.6)	0.09	4173.0 (11.5)	29224.5 (11.9)	0.02

[a]Missing values for race/ethnicity, VA priority group, driving distance to nearest VA facility, census region, rurality generated using single imputation
[b]Descriptive statistics and SMD after applying inverse probability of treatment weight
[c]SMD for categorical variables with >2 levels represented using sets of binary indicator variables (34)
[d]Veterans are assigned to 1 of 8 priority groups at VA enrollment based on service-connected illnesses, era of Service, and socioeconomic status determined by means testing. Priority group determines level of co-payment.
[e]The complexity rating is based on a VAMC's patient volume, number and breadth of physician specialists, patient case mix, intensive care unit capabilities, and degree of teaching and research.

© 2022 Pickering AN et al. *JAMA Network Open*

eTable 3. Standardized Mean Differences for Baseline Patient- and Facility-Level Characteristics Between Veterans Who Underwent Low-Value Prostate-Specific Antigen Testing Within Medicare and Those Who Did Not Undergo Low-Value PSA Testing Before and After Inverse Probability of Treatment Weighting[a]

	Unweighted			Weighted[b]		
	**Medicare PSA Group (n=17,981)**	**Control Group (n=245,953)**	**SMD[c]**	**Medicare PSA Group (n=17,981)**	**Control Group (n=245,953)**	**SMD[c]**
**Patient-level characteristics**						
Age, y, mean (SD)	81.3 (5.2)	83.1 (5.6)	0.33	83.2 (5.7)	83.0 (5.6)	0.03
Race/ethnicity, n (%)						
Hispanic	464 (2.6)	7126 (2.9)	0.02	530.6 (3.0)	7073.0 (2.9)	0.00
Non-Hispanic black	1055 (5.9)	16426 (6.7)	0.04	1184.7 (6.6)	16289.1 (6.6)	0.00
Non-Hispanic other + Multiracial	423 (2.4)	5853 (2.4)	0.00	435.4 (2.4)	5848.8 (2.4)	0.00
Non-Hispanic white	16039 (89.2)	216548 (88.0)	ref	15830.2 (88.0)	216742.2 (88.1)	ref
VA priority group[d], n (%)						
1	3500 (19.5)	46470 (18.9)	ref	3312.1 (18.4)	46559.5 (18.9)	ref
2	1288 (17.2)	14590 (5.9)	0.07	1047.3 (5.8)	14793.7 (6.0)	0.00
3	2458 (13.7)	27851 (11.3)	0.08	2003.4 (11.1)	28240.2 (11.5)	0.00
4	282 (1.6)	7777 (3.2)	0.22	576.8 (3.2)	7510.8 (3.1)	0.03
5	2355 (13.1)	56590 (23.0)	0.30	4095.3 (22.8)	54931.7 (22.30	0.02
6	550 (3.1)	3754 (1.5)	0.20	283.5 (1.6)	4009.2 (1.6)	0.00
7	828 (4.6)	10588 (4.3)	0.01	802.3 (4.5)	10639.7 (4.3)	0.02
8	6720 (37.4)	78333 (31.9)	0.06	5860.4 (32.6)	79268.3 (32.2)	0.02
Driving distance to nearest VA facility, miles, mean (SD)	16.6 (15.8)	16.8 (16.6)	0.02	17.2 (17.0)	16.8 (16.6)	0.02
Number of Elixhauser conditions, mean (SD)	3.0 (2.2)	2.8 (2.3)	0.07	2.9 (2.3)	2.8 (2.3)	0.03
HIV/AIDS, n (%)	11 (0.1)	207 (0.1)	0.01	14.1 (0.1)	203.0 (0.1)	0.00
Alcohol use disorder	194 (1.1)	3256 (1.3)	0.02	238.6 (1.3)	3215.4 (1.3)	0.00
Iron-deficiency anemia	3246 (18.1)	40747 (16.6)	0.04	3075.1 (17.1)	41000.5 (16.7)	0.01
Rheumatoid/collagen-vascular disease	740 (4.1)	7496 (3.1)	0.06	574.7 (3.2)	7676.7 (3.1)	0.00

© 2022 Pickering AN et al. *JAMA Network Open*

	Unweighted			Weighted[b]		
	Medicare PSA Group (n=17,981)	Control Group (n=245,953)	SMD[c]	Medicare PSA Group (n=17,981)	Control Group (n=245,953)	SMD[c]
**Patient-level characteristics**						
Blood loss anemia	197 (1.1)	2557 (1.0)	0.01	183.2 (1.0)	2566.5 (1.0)	0.00
Congestive heart failure	2273 (12.6)	37165 (15.1)	0.07	2851.6 (15.9)	36759.8 (15.0)	0.03
Chronic pulmonary disease	3678 (20.5)	49576 (20.2)	0.01	3757.0 (20.9)	49634.7 (20.2)	0.02
Coagulation deficiency	761 (4.2)	9298 (3.8)	0.02	696.6 (3.9)	9374.1 (3.8)	0.00
Depression	1434 (8.0)	20650 (8.4)	0.02	1559.0 (8.7)	20582.0 (8.4)	0.01
Diabetes without chronic conditions	2486 (13.8)	31515 (12.8)	0.03	2291.8 (12.8)	31682.5 (12.9)	0.00
Diabetes with chronic conditions	4213 (23.4)	55320 (22.5)	0.02	4118.1 (22.9)	55482.1 (22.6)	0.01
Drug abuse	65 (0.4)	838 (0.3)	0.00	61.5 (0.3)	841.7 (0.3)	0.00
Uncomplicated hypertension	11206 (62.3)	132740 (54.0)	0.17	9819.4 (54.6)	134141.2 (54.5)	0.00
Complicated hypertension	3122 (17.4)	43349 (17.6)	0.01	3271.3 (18.2)	43308.1 (17.6)	0.02
Hypothyroidism	2999 (16.7)	32124 (13.1)	0.10	2459.7 (13.7)	32736.7 (13.3)	0.01
Liver disease	330 (1.8)	3824 (1.6)	0.02	261.3 (1.5)	3868.7 (1.6)	0.01
Lymphoma	231 (1.3)	2982 (1.2)	0.01	222.3 (1.2)	2994.9 (1.2)	0.00
Fluid and electrolyte disorder	1593 (8.9)	23796 (9.7)	0.03	1836.6 (10.2)	23665.3 (9.6)	0.02
Metastatic cancer	94 (0.5)	1454 (0.6)	0.01	109.8 (0.6)	1443.0 (0.6)	0.00
Other neurologic disorder	1804 (10.0)	28685 (11.7)	0.05	2198.2 (12.3)	28417.4 (11.6)	0.02
Obesity	2168 (12.1)	21019 (8.6)	0.12	1586.7 (8.8)	21606.9 (8.8)	0.00
Paralysis	279 (1.6)	4420 (1.8)	0.02	354.7 (2.0)	4379.9 (1.8)	0.02
Peripheral vascular disease	3261 (18.1)	39846 (16.2)	0.05	3053.6 (17.0)	40179.3 (16.3)	0.02
Psychosis	313 (1.7)	4659 (1.9)	0.01	348.6 (1.9)	4633.8 (1.9)	0.00
Pulmonary circulation disorder	230 (1.3)	3594 (1.5)	0.02	266.2 (1.5)	3564.2 (1.5)	0.00
Renal failure	3187 (17.7)	46073 (18.7)	0.03	3414.7 (19.0)	45905.8 (18.7)	0.01
Solid tumor without metastasis	658 (3.7)	8702 (3.5)	0.01	619.1 (3.4)	8721.9 (3.6)	0.01

© 2022 Pickering AN et al. *JAMA Network Open*

	Unweighted			Weighted[b]		
	**Medicare PSA Group (n=17,981)**	**Control Group (n=245,953)**	**SMD[c]**	**Medicare PSA Group (n=17,981)**	**Control Group (n=245,953)**	**SMD[c]**
**Patient-level characteristics**						
Peptic ulcer disease	135 (99.3)	1978 (0.8)	0.01	142.3 (0.8)	1969.5 (0.8)	0.00
Valvular disease	2180 (12.1)	27728 (11.3)	0.03	2107.3 (11.7)	27875.4 (11.3)	0.01
Weight loss	393 (2.2)	7586 (3.1)	0.06	587.4 (3.30	7437.1 (3.1)	0.01
**Facility-level characteristics**						
Academic affiliation, yes, n (%)	17719 (98.5)	240547 (97.8)	0.06	17605.6 (97.9)	240671.2 (97.9)	0.00
Facility size, total # outpatient visits FY18, mean (SD)	788769 (419935)	740286 (395187)	0.12	741462.5 (397585.7)	743574.1 (397094.6)	0.01
Census region, n (%)						
Northeast	2917 (16.2)	41194 (16.8)	ref	2964.3 (16.5)	41103.2 (16.7)	ref
Midwest	3812 (21.2)	61568 (25.0)	0.07	4514.3 (25.1)	60930.0 (24.8)	0.01
South	8505 (47.3)	96544 (39.3)	0.10	7199.4 (40.0)	97894.2 (39.8)	0.01
West	2747 (15.3)	46647 (19.0)	0.09	3303.1 (18.4)	46025.6 (18.7)	0.00
Rurality, n (%)						
Large metropolitan	6685 (37.3)	84832 (34.5)	ref	6291.9 (35.0)	85398.1 (34.7)	ref
Small metropolitan	7031 (39.2)	93055 (37.9)	0.02	6687.6 (37.20	93423.8 (38.0)	0.01
Micropolitan	2441 (13.6)	38005 (15.5)	0.09	2804.1 (15.6)	37743.0 (15.4)	0.00
Noncore rural	1789 (10.0)	29713 (12.1)	0.11	2197.4 (12.2)	29388.1 (12.0)	0.01
Facility complexity level[e], n (%)						
High complexity	14161 (78.8)	188152 (76.5)	ref	13752.0 (76.5)	188527.0 (76.7)	ref
Medium complexity	1859 (10.3)	29385 (12.0)	0.06	2130.0 (11.9)	29117.3 (11.8)	0.00
Low complexity	1961 (10.9)	28416 (11.6)	0.03	2099.0 (11.7)	28308.7 (11.5)	0.01

[a]Missing values for race/ethnicity, VA priority group, driving distance to nearest VA facility, census region, rurality generated using single imputation
[b]Descriptive statistics and SMD after applying inverse probability of treatment weight
[c]SMD for categorical variables with >2 levels represented using sets of binary indicator variables (34)
[d]Veterans are assigned to 1 of 8 priority groups at VA enrollment based on service-connected illnesses, era of Service, and socioeconomic status determined by means testing. Priority group determines level of co-payment.
[e]The complexity rating is based on a VAMC's patient volume, number and breadth of physician specialists, patient case mix, intensive care unit capabilities, and degree of teaching and research.

© 2022 Pickering AN et al. *JAMA Network Open*

eTable 4. Standardized Mean Differences for Baseline Patient- and Facility-Level Characteristics Between Veterans Who Underwent Low-Value Prostate-Specific Antigen Testing Within the US Veterans Health Administration and Medicare Before and After Inverse Probability of Treatment Weighting[a]

	**Unweighted**			**Weighted[b]**		
	**VHA PSA Group (n=36,459)**	**Medicare PSA Group (n=17,981)**	**SMD[c]**	**VHA PSA Group (n=36,459)**	**Medicare PSA Group (n=17,981)**	**SMD[c]**
**Patient-level characteristics**						
Age, y, mean (SD)	80.0 (4.7)	81.3 (5.2)	0.28	80.5 (5.0)	80.5 (4.8)	0.01
Race/ethnicity, n (%)						
Hispanic	1170 (3.2)	464 (2.6)	0.04	1093.4 (3.0)	537.7 (3.0)	0.00
Non-Hispanic black	2653 (7.3)	1055 (5.9)	0.06	2479.6 (6.8)	1216.4 (6.8)	0.00
Non-Hispanic other + Multiracial	812 (2.2)	423 (2.4)	0.01	823.2 (2.3)	403.3 (2.2)	0.00
Non-Hispanic white	31824 (87.3)	16039 (89.2)	ref	32062.7 (87.9)	15823.5 (88.0)	ref
VA priority group[d], n (%)						
1	7716 (21.2)	3500 (19.5)	ref	7518.3 (20.6)	3728.6 (20.7)	ref
2	2247 (6.2)	1288 (17.2)	0.10	2368.3 (6.5)	1179.5 (6.6)	0.00
3	4389 (12.0)	2458 (13.7)	0.10	4610.6 (12.7)	2310.9 (12.9)	0.01
4	626 (1.7)	282 (1.6)	0.00	607.1 (1.7)	304.4 (1.7)	0.00
5	8369 (23.0)	2355 (13.1)	0.24	7154.7 (19.6)	3470.2 (19.3)	0.01
6	866 (2.4)	550 (3.1)	0.11	941.9 (2.6)	466.4 (2.6)	0.00
7	1155 (3.2)	828 (4.6)	0.17	1338.5 (3.7)	670.8 (3.7)	0.00
8	11091 (30.4)	6720 (37.4)	0.14	11919.6 (32.7)	5850.2 (32.5)	0.01
Driving distance to nearest VA facility, miles, mean (SD)	18.8 (18.8)	16.6 (15.8)	0.13	18.0 (18.1)	18.0 (17.3)	0.00
Number of Elixhauser conditions, mean (SD)	2.3 (2.1)	3.0 (2.2)	0.32	2.5 (2.2)	2.6 (2.1)	0.02
HIV/AIDS, n (%)	37 (0.1)	11 (0.1)	0.01	32.3 (0.1)	16.8 (0.1)	0.00
Alcohol use disorder	446 (1.2)	194 (1.1)	0.01	429.5 (1.2)	214.6 (1.2)	0.00
Iron-deficiency anemia	4092 (11.2)	3246 (18.1)	0.19	4974.9 (13.7)	2494.4 (13.9)	0.01
Rheumatoid/collagen-vascular disease	1018 (2.8)	740 (4.1)	0.07	1193.2 (3.3)	601.2 (3.3)	0.00

© 2022 Pickering AN et al. *JAMA Network Open*

	Unweighted			Weighted[b]		
	VHA PSA Group (n=36,459)	Medicare PSA Group (n=17,981)	SMD[c]	VHA PSA Group (n=36,459)	Medicare PSA Group (n=17,981)	SMD[c]
**Patient-level characteristics**						
Blood loss anemia	240 (0.7)	197 (1.1)	0.05	300.0 (0.8)	150.0 (0.8)	0.00
Congestive heart failure	3602 (9.9)	2273 (12.6)	0.09	3970.0 (10.9)	2004.7 (11.2)	0.01
Chronic pulmonary disease	6312 (17.3)	3678 (20.5)	0.08	6719.2 (18.4)	3378.2 (18.8)	0.01
Coagulation deficiency	928 (2.6)	761 (4.2)	0.09	1135.1 (3.1)	571.4 (3.2)	0.00
Depression	2665 (7.3)	1434 (8.0)	0.03	2764.4 (7.6)	1400.4 (7.8)	0.01
Diabetes without chronic conditions	4867 (13.4)	2486 (13.8)	0.01	4915.4 (13.5)	2426.5 (13.5)	0.00
Diabetes with chronic conditions	7064 (19.4)	4213 (23.4)	0.10	7596.5 (20.8)	3792.1 (21.1)	0.01
Drug abuse	151 (0.4)	65 (0.4)	0.01	146.0 (0.4)	74.2 (0.4)	0.00
Uncomplicated hypertension	18699 (51.3)	11206 (62.3)	0.22	20031.3 (54.9)	9958.9 (55.4)	0.01
Complicated hypertension	4589 (12.6)	3122 (17.4)	0.13	5227.6 (14.3)	2642.9 (14.7)	0.01
Hypothyroidism	3836 (10.5)	2999 (16.7)	0.18	4634.8 (12.7)	2337.0 (13.0)	0.01
Liver disease	516 (1.4)	330 (1.8)	0.03	566.5 (1.6)	277.6 (1.5)	0.00
Lymphoma	389 (1.1)	231 (1.3)	0.02	426.0 (1.2)	217.6 (1.2)	0.00
Fluid and electrolyte disorder	2416 (6.6)	1593 (8.9)	0.08	2709.3 (7.4)	1366.4 (7.6)	0.01
Metastatic cancer	150 (0.4)	94 (0.5)	0.02	168.2 (0.5)	86.4 (0.5)	0.00
Other neurologic disorder	2842 (7.8)	1804 (10.0)	0.08	3135.7 (8.6)	1568.9 (8.7)	0.00
Obesity	3147 (8.6)	2168 (12.1)	0.11	3595.9 (9.9)	1804.7 (10.0)	0.01
Paralysis	491 (1.4)	279 (1.6)	0.02	510.7 (1.4)	256.1 (1.4)	0.00
Peripheral vascular disease	4327 (11.9)	3261 (18.1)	0.18	5133.1 (14.1)	2557.0 (14.2)	0.00
Psychosis	600 (1.7)	313 (1.7)	0.01	608.8 (1.7)	302.1 (1.7)	0.00
Pulmonary circulation disorder	415 (1.1)	230 (1.3)	0.01	441.1 (1.2)	266.5 (1.3)	0.00
Renal failure	4586 (12.6)	3187 (17.7)	0.14	5279.8 (14.5)	2641.8 (14.7)	0.01
Solid tumor without metastasis	1140 (3.1)	658 (3.7)	0.03	1215.1 (3.3)	602.8 (3.4)	0.00

© 2022 Pickering AN et al. *JAMA Network Open*

	Unweighted			Weighted[b]		
	VHA PSA Group (n=36,459)	Medicare PSA Group (n=17,981)	SMD[c]	VHA PSA Group (n=36,459)	Medicare PSA Group (n=17,981)	SMD[c]
**Patient-level characteristics**						
Peptic ulcer disease	224 (0.6)	135 (99.3)	0.02	241.8 (0.7)	120.2 (0.7)	0.00
Valvular disease	2910 (8.0)	2180 (12.1)	0.14	3447.4 (9.5)	1733.2 (9.6)	0.01
Weight loss	688 (1.9)	393 (2.2)	0.02	735.7 (2.0)	377.6 (2.1)	0.01
**Facility-level characteristics**						
Academic affiliation, yes, n (%)	35663 (97.8)	17719 (98.5)	0.05	35754.8 (98.1)	17641.2 (98.1)	0.00
Facility size, total # outpatient visits FY18, mean (SD)	755774 (437479)	788769 (419935)	0.08	768551.6 (437350.3)	772056.5 (421867.8)	0.01
Census region, n (%)						
Northeast	3502 (9.6)	2917 (16.2)	ref	4333.5 (11.9)	2128.4 (11.8)	ref
Midwest	8402 (23.1)	3812 (21.2)	0.29	8174.0 (22.4)	4056.6 (22.6)	0.00
South	17752 (48.7)	8505 (47.3)	0.22	17560.0 (48.3)	8736.4 (48.6)	0.00
West	6803 (18.7)	2747 (15.3)	0.36	6351.6 (17.4)	3059.7 (17.0)	0.01
Rurality, n (%)						
Large metropolitan	9938 (27.3)	6685 (37.3)	ref	11151.4 (30.6)	5489.7 (30.5)	ref
Small metropolitan	14582 (40.0)	7031 (39.2)	0.17	14499.9 (39.8)	7143.0 (39.7)	0.00
Micropolitan	6222 (17.1)	2441 (13.6)	0.25	5811.7 (15.9)	2890.6 (16.1)	0.00
Noncore rural	5687 (15.6)	1789 (10.0)	0.34	4995.9 (13.7)	2457.8 (13.7)	0.00
Facility complexity level[e], n (%)						
High complexity	26709 (73.3)	14161 (78.8)	ref	27407.2 (75.2)	13547.1 (75.3)	ref
Medium complexity	4587 (12.6)	1859 (10.3)	0.09	4307.5 (11.8)	2107.4 (11.7)	0.00
Low complexity	5163 (14.2)	1961 (10.9)	0.12	4744.3 (13.0)	2326.5 (12.9)	0.00

[a]Missing values for race/ethnicity, VA priority group, driving distance to nearest VA facility, census region, rurality generated using single imputation
[b]Descriptive statistics and SMD after applying inverse probability of treatment weight
[c]SMD for categorical variables with >2 levels represented using sets of binary indicator variables[34]
[d]Veterans are assigned to 1 of 8 priority groups at VA enrollment based on service-connected illnesses, era of Service, and socioeconomic status determined by means testing. Priority group determines level of co-payment.
[e]The complexity rating is based on a VAMC's patient volume, number and breadth of physician specialists, patient case mix, intensive care unit capabilities, and degree of teaching and research.

© 2022 Pickering AN et al. *JAMA Network Open*

eTable 5. Difference in Use of Potential Cascade Services in Veterans Aged Older than 83 Years Who Received Low-Value Prostate-Specific Antigen Testing Within the US Veterans Health Administration Versus Those Who Did Not Receive Low-Value PSA Testing

**Cascade services**	**Unadjusted Rate per 100 Veterans**		**Unadjusted Difference in Rate per 100 Veterans**[a]	**Adjusted Difference in rate per 100 Veterans (95% CI)**[b]
	VHA PSA Group (n=8,211)	Control Group (n=110,956)		
Total cascade services	46.9	12.9	34.0	32.8 (29.9-35.8)
Additional PSA test	16.6	3.2	13.0	12.8 (11.3-14.3)
Related outpatient visit[c]	12.3	1.5	10.8	10.6 (8.9-12.2)
Urology visit	15.5	7.8	7.7	7.6 (5.9-9.3)
Prostate imaging	1.2	0.4	0.8	0.7 (0.5-1.0)
Prostate biopsy	0.2	0.02	0.2	0.2 (0.1-0.3)
Androgen deprivation therapy	0.7	0.04	0.7	0.6 (0.3-0.9)
Surgical procedure[d]	<0.2[e]	<0.2[e]	<0.2[e]	-
Radiation therapy[f]	0.4	0.04	0.4	0.3 (-0.1-0.7)

VHA, Veterans Health Administration

[a]Determined by subtracting rate of potential cascade services per 100 Veterans in those who did not receive low-value PSA testing from those who received low-value PSA within VHA

[b]Adjusted for patient and VA Medical Center-level covariates (age, race/ethnicity, VA priority group, driving distance to nearest VA, number of Elixhauser conditions, individual Elixhauser conditions, academic affiliation, facility size, census region, rurality, complexity level) using inverse probability of treatment weighting

[c]Outpatient visit or consultation with primary diagnosis of prostate cancer or elevated PSA

[d]Prostatectomy or surgical resection of prostate.

[e]Cell size suppressed due to data use agreements

[f]Contact radiation, beam radiation, insertion of radioactive element, brachytherapy, intraoperative radiation therapy, plaque radiation of prostate

© 2022 Pickering AN et al. *JAMA Network Open*

eTable 6. Difference in Use of Potential Cascade Services in Veterans Aged Older than 83 Years Who Received Low-Value Prostate-Specific Antigen Testing Within Medicare Versus Those Who Did Not Receive Low-Value PSA Testing

**Cascade services**	**Unadjusted Rate per 100 Veterans**		**Unadjusted Difference in Rate per 100 Veterans**[a]	**Adjusted Difference in rate per 100 Veterans (95% CI)**[b]
	Medicare PSA Group (n=5,787)	Control Group (n=110,956)		
Total cascade services	51.6	12.9	38.7	38.5 (35.2-41.7)
Additional PSA test	15.4	3.2	12.2	11.9 (10.8-13.0)
Related outpatient visit[c]	9.1	1.5	7.6	8.0 (6.6-9.4)
Urology visit	25.1	7.8	17.3	16.8 (15.3-18.4)
Prostate imaging	0.3	0.4	-0.1	-0.09 (-0.17- -0.01)
Prostate biopsy	0.5	0.02	0.5	0.5 (0.3-0.8)
Androgen deprivation therapy	0.8	0.04	0.7	0.9 (0.4-1.4)
Surgical procedure[d]	<0.2[e]	<0.2[e]	<0.2[e]	-
Radiation therapy[f]	0.4	0.04	0.4	0.36 (0.04-0.67)

[a]Determined by subtracting rate of potential cascade services per 100 Veterans in those who did not receive low-value PSA testing from those who received low-value PSA within Medicare
[b]Adjusted for patient and VA Medical Center-level covariates (age, race/ethnicity, VA priority group, driving distance to nearest VA, number of Elixhauser conditions, individual Elixhauser conditions, academic affiliation, facility size, census region, rurality, complexity level) using inverse probability of treatment weighting
[c]Outpatient visit or consultation with primary diagnosis of prostate cancer or elevated PSA
[d]Prostatectomy or surgical resection of prostate.
[e]Cell size suppressed due to data use agreements
[f]Contact radiation, beam radiation, insertion of radioactive element, brachytherapy, intraoperative radiation therapy, plaque radiation of prostate

© 2022 Pickering AN et al. *JAMA Network Open*

eTable 7. Difference in Use of Potential Cascade Services in Veterans Aged Older than 83 Years Who Received Low-Value Prostate-Specific Antigen Testing Within Medicare Versus Those Who Received Low-Value PSA Testing Within the US Veterans Health Administration

Cascade services	Unadjusted Rate per 100 Veterans		Unadjusted Difference in Rate per 100 Veterans[a]	Adjusted Difference in rate per 100 Veterans (95% CI)[b]
	Medicare PSA Group (n=5,787)	VHA PSA Group (n=8,211)		
Total cascade services	51.6	46.9	4.7	5.6 (5.2-6.0)
Additional PSA test	15.4	16.6	-1.2	-1.0 (-0.8- -1.1)
Related outpatient visit[c]	9.1	12.3	-3.2	-2.6 (-2.7- -2.6)
Urology visit	25.1	15.5	9.6	9.5 (9.3-9.6)
Prostate imaging	0.3	1.2	-0.9	-0.9 (-0.7- -1.0)
Prostate biopsy	0.5	0.2	0.3	0.3 (0.2-0.5)
Androgen deprivation therapy	0.8	0.7	0.1	0.2 (0.1-0.4)
Surgical procedure[d]	<0.2[e]	<0.2[e]	<0.2[e]	-
Radiation therapy[f]	0.43	0.35	0.1	0.04 (-0.08-0.16)

VHA, Veterans Health Administration

[a]Determined by subtracting rate of potential cascade services per 100 Veterans in those who received low-value PSA within VHA from those who received low-value PSA testing within Medicare

[b]Adjusted for patient and VA Medical Center-level covariates (age, race/ethnicity, VA priority group, driving distance to nearest VA, number of Elixhauser conditions, individual Elixhauser conditions, academic affiliation, facility size, census region, rurality, complexity level) using inverse probability of treatment weighting

[c]Outpatient visit or consultation with primary diagnosis of prostate cancer or elevated PSA

[d]Prostatectomy or surgical resection of prostate.

[e]Cell size suppressed due to data use agreements

[f]Contact radiation, beam radiation, insertion of radioactive element, brachytherapy, intraoperative radiation therapy, plaque radiation of prostate

© 2022 Pickering AN et al. *JAMA Network Open*

eTable 8. Rates of Potential Cascade Services After Low-Value Prostate-Specific Antigen Testing by Health Care System

	**Location of index PSA test**			
	**VHA PSA Group (n=36,459)**		**Medicare PSA Group (n=17,981)**	
	Unadjusted rate per 100 Veterans			
Location of care cascade service	*VHA*	*Medicare*	*VHA*	*Medicare*
Total cascade services	35.0	10.2	13.8	40.1
Subsequent PSA test	15.8	2.9	10.6	9.7
Related outpatient visit[a]	8.5	1.5	1.8	6.3
Urology visit	8.9	5.3	1.2	22.0
Prostate imaging	1.2	<0.1[b]	0.2	0.1
Prostate biopsy	0.3	0.2	<0.1[b]	0.7
Androgen deprivation therapy	0.3	0.1	<0.1[b]	0.5
Surgical procedure[c]	<0.1[b]	<0.1[b]	<0.1[b]	<0.1[b]
Radiation therapy[d]	0.1	0.2	<0.1[b]	0.8

VHA, Veterans Health Administration
[a]Outpatient visit or consultation with primary diagnosis of prostate cancer or elevated PSA
[b]Cell size suppressed due to Medicare data use agreement
[c]Prostatectomy or surgical resection of prostate
[d]Contact radiation, beam radiation, insertion of radioactive element, brachytherapy, intraoperative radiation therapy, plaque radiation of prostate

© 2022 Pickering AN et al. *JAMA Network Open*
