# Supplementary material for: Assessment of Care Cascades Following Low-Value Prostate-Specific Antigen Testing Among Veterans Dually Enrolled in the US Veterans Health Administration and Medicare Systems
Source: JAMA Netw Open. 2022 Dec 15;5(12):e2247180. doi: 10.1001/jamanetworkopen.2022.47180 (PMC9856440; doi:10.1001/jamanetworkopen.2022.47180)
Supplement: Supplementary file 1 [file jamanetwopen-e2247180-s002.pdf]

Data Sharing Statement

Pickering. Assessment of Care Cascades Following Low-Value Prostate-Specific Antigen Testing Among Veterans Dually Enrolled in the US Veterans Health Administration and Medicare Systems. *JAMA Netw Open*. Published December 15, 2022. doi:10.1001/jamanetworkopen.2022.47180

Data
Data available: No
